# Analysis of risk and prognostic factors in a population of pediatric patients hospitalized for acute malnutrition at the Chiulo hospital, Angola

**DOI:** 10.1186/s13052-021-01140-2

**Published:** 2021-09-10

**Authors:** Federica Maria Tripoli, Salvatore Accomando, Simona La Placa, Andrea Pietravalle, Giovanni Putoto, Giovanni Corsello, Mario Giuffrè

**Affiliations:** 1 Department of Health Promotion, Mother and Child Care, Internal Medicine and Medical Specialties, University of Palermo, Palermo, Italy; 2 Doctors with Africa, CUAMM, Chiulo, Ombadja, Angola

**Keywords:** Severe acute malnutrition, Wasting, Risk factors, Prognostic factors, Sub-Saharan Africa, Breastfeeding

## Abstract

**Background:**

Malnutrition is a multifactorial pathology in which genetic, epigenetic, cultural, environmental, socio-economic factors interact with each other. The impact that this disease has on the health of children worldwide is dramatic. Severe acute malnutrition in particular is a disease affecting nearly 20 million preschool children worldwide, most of them in Africa and South East Asia.

**Objectives:**

This work aims to investigate potential prognostic factors in the clinical evolution of acute malnutrition and potential risk factors for the development of the disease.

**Methods:**

Our study was carried out at the “Hospital da Missão Catolica do Chiulo”, in Angola, where the NGO Doctors with Africa CUAMM has been operating since 2000. In the first part of the study we analyzed the characteristics and clinical evolution of 163 patients hospitalized for acute malnutrition at the UEN (Unidade Especial de Nutrição) of the Chiulo Hospital over a period of 6 months, in order to identify potential prognostic factors of the disease. The second part of our study was carried out by administering a questionnaire to a group of caregivers of malnourished children and to a group of caregivers of non-malnourished children admitted to Pediatrics for other causes, with the aim of identifying potential risk factors for the development of malnutrition.

**Results and conclusions:**

The analysis of prognostic factors revealed that the most relevant are the WHZ (weight for height z-score) at the time of admission, the presence of Stunting and the presence of other pathologies or clinical conditions associated with severe acute malnutrition.

The analysis of risk factors has shown that not only food shortages, but also errors in the timing of the suspension of breastfeeding and the timing of the introduction of complementary foods play an important role. Equally important were some family risk factors, including the size of the family unit and the presence of deceased children.

It also emerged that the lack of knowledge of what a child needs to grow up healthy often affects the development of malnutrition. It follows that a useful and low-cost tool for preventing child malnutrition would be large-scale nutrition education campaigns.

## Background

Doctors with Africa CUAMM is an NGO that works to ensuring the right to health and to make access to health services available to everyone. It is active today in eight countries of sub-Saharan Africa with long-term health care projects, including Angola were this study was carried out [1].

Angola is a country with a high fertility rate (5.6 births per woman of childbearing age) and a population growth rate of 2.7% per year [2]. The infant mortality rate is 44 deaths under the first year of life for every 1000 live births and under 5 mortality is 68 for every 1000 live births. 30% of the Angolan population and 58% of that of rural areas lives below the poverty line [3]. In a context such as the one described, malnutrition among childhood pathologies, is certainly one of the most widespread. In Angola the prevalence of moderate chronic malnutrition under the age of 5 is 38%, the prevalence of severe chronic malnutrition is 15%. The percentage of children under 5 who suffer from moderate acute malnutrition is 5%, the percentage of those who suffer from a severe acute form is 1%.

The province of Cunene, where this study was carried out, is the one with the highest percentage of acute malnutrition (11% of children under 5 years). It is estimated that only 13% of children between 6 and 23 months meet the WHO criteria for minimum acceptable diet (only 2% In the provinces of Luanda Norte and Cunene [3].

Our study took place at the “Hospital da Missão Catolica do Chiulo”, owned by the Catholic diocese of Ondjiva but also supported by the Angolan government and CUAMM. Its reference population is about 300,000 people organized in family groups and residing in rural areas without telephone networks, public transport, electricity and water [1].

There are three main forms of malnutrition: undernutrition, hidden hunger and overweight [4].

Our work has focused on the study of childhood undernutrition. In the following discussion, for simplicity, the term “malnutrition” will be used to refer to the condition of undernutrition.

Malnutrition is both a consequence and one of the main causes of poverty and deprivation. The impairment of the physical and cognitive development of citizens, inevitably, has repercussions on the development of the entire country. A state of malnutrition, especially in the first thousand days, has significant consequences. In fact, it is a crucial period for the establishment of proper linear growth and adequate neurological development.

In the context of undernutrition, we can distinguish at least three different clinical conditions with reference to 2006 WHO growth charts:

- “Stunting”, term that indicates the state of chronic malnutrition, defined by a height/length for age z-score less than − 2 SD. It is an expression of linear growth retardation.
- “Wasting”, term that indicate s the state of acute malnutrition, defined by a weight for height/length z-score less than − 2 SD. This index is an expression of body mass in relation to height and describes the current nutritional status. It is further distinguished into SAM (Severe Acute Malnutrition) when the WHZ is <−3SD, and MAM (Moderate Acute Malnutrition) when the WHZ is between − 2 SD and -3SD.
- “Underweight**”**, a composite index that takes into account both chronic malnutrition and acute malnutrition but does not distinguish between the two and which is defined by a weight for age z-score less than − 2 SD [5].

So, it is essential for the diagnosis and clinical management of malnutrition, to get accurate anthropometric measurements and therefore proper tools and specifically trained personnel. This can be a challenge, especially in rural contexts [6]. It is estimated that SAM affects nearly 20 million preschool children around the world, most of them are in Africa and South East Asia [7]. Globally, acute malnutrition triggers more than 50% of childhood mortality in children under 5 years old, which implies that about 3.5 million children die of malnutrition each year [8, 9].

According to the WHO guidelines of 2013 (the latest published), the diagnosis of Severe Acute Malnutrition is placed in the presence of at least one of the following three criteria: 1) weight for height/lenght z-score < − 3 SD; 2) MUAC < 11,5 cm in children between 6 and 59 months; 3) presence of bilateral pitting edema.

The diagnosis of SAM imposes the need to include the patient in a program for clinical management and follow-up of malnutrition. First, it will be established whether the patient’s condition requires hospitalization and consequent “intensive” treatment or an outpatient management may be sufficient, ideally in the health center closest to home [10].

## Objectives

The objective of this study is to investigate potential prognostic factors in the clinical evolution of acute malnutrition and potential risk factors for the development of this disease, by studying a low-resource hospital setting in a country with poor health indicators.

## Patients and methods

Our study consists of two parts. In the first one, we analyzed a sample of 163 patients admitted to the UEN (Unidade Especial de Nutrição) of the Chiulo Hospital for acute malnutrition, over a period of 6 months (November 2018–May 2019). For each patient we collected data on age, sex, anthropometric data at entry (weight, length, MUAC, weight for height z-score, weight for age z-score, height for age z-score), presence or absence of edema, anthropometric data at discharge, outcome (discharged, escaped or deceased), average weight gain (g/Kg/die), length of hospitalization and presence of pathologies or associated clinical conditions. The goal of this first part was to identify possible prognostic factors in the clinical evolution of the disease. The analysis was carried out by dividing the patients into groups on the basis of some variables, considered as potential prognostic factors (age, sex, severity of malnutrition, presence of stunting, presence of associated pathologies), and comparing the different groups in relation to the following clinical outcomes: length of hospitalization, average weight gain, difference between discharge weight and target weight (weight for which WHZ is − 2 DS), difference between MUAC at admission and at discharge and mortality. The averages of the parameters evaluated as outcomes, have been calculated without considering the patients who died and escaped, so that these data were not influenced by mortality. In the comparison between the different groups, the evaluation of statistical significance was carried out by applying the Student’s T test.

**Fig. 1:**
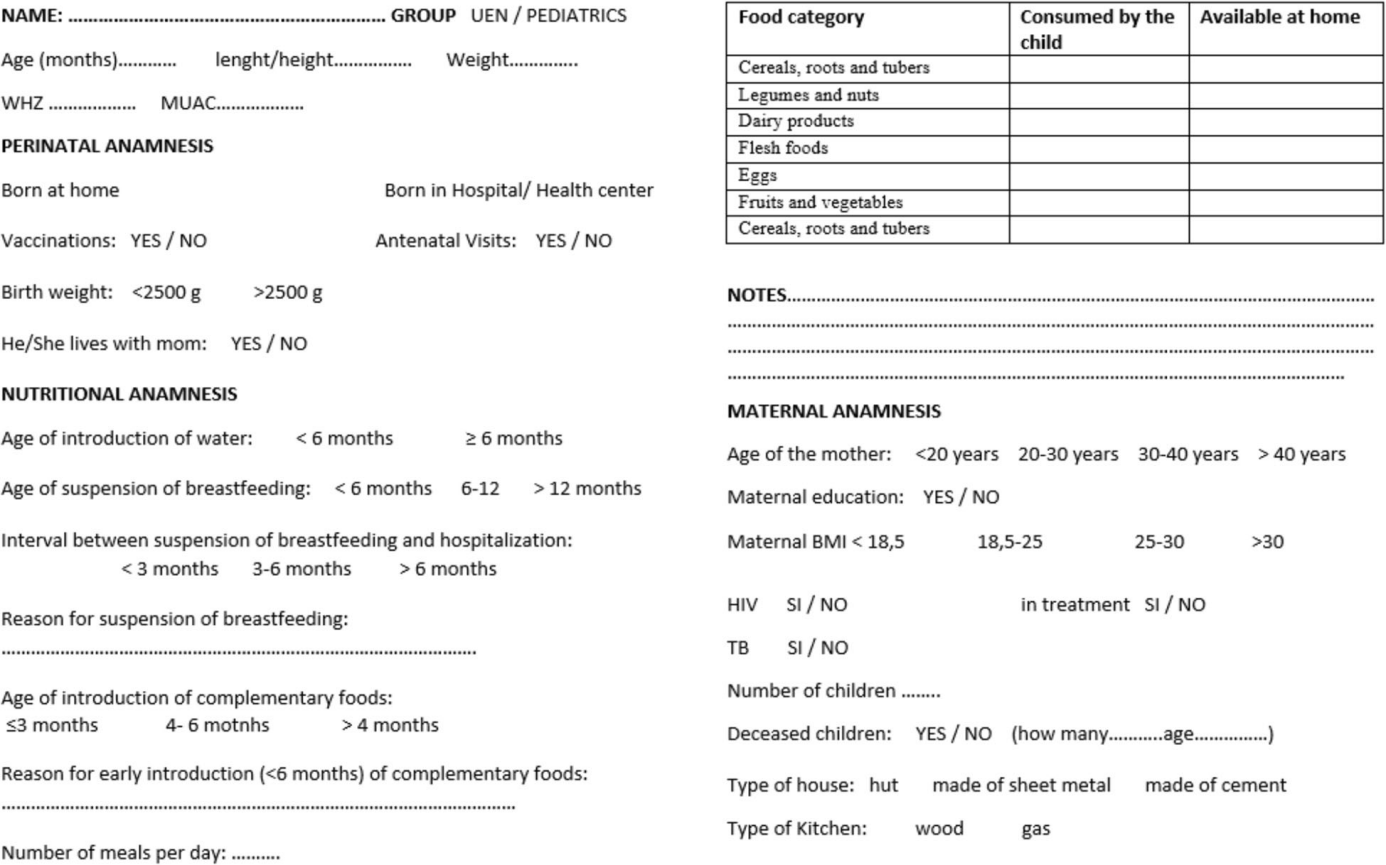
Questionnaire on risk factors for malnutrition

The second part of our study was carried out by administering a questionnaire to a group of malnourished children’s caregivers and to a group of caregivers of non-malnourished children admitted to Pediatrics for other causes. The questionnaire consisted of a first part with the patient’s personal and anthropometric data, a section on perinatal anamnesis, with particular attention to the opportunities of access to health services, then the nutritional anamnesis, the maternal anamnesis and some data relating to socio-economic conditions of the family nucleus (Fig. 1). The goal of this second part of the study was to identify possible risk factors for the development of acute malnutrition. The questionnaires were administered to the caregivers with the help of Angolan nurses who acted as interpreters, translating the questions from Portuguese to the local dialect to make them understandable to mothers who did not speak Portuguese (most of them). Each questionnaire took about 20 min, making that moment an opportunity to perform nutritional education. Given the time required for administration, and the need for local staff available for translation, the final number of questionnaires was limited (52 mothers of malnourished children and 22 mothers of non-malnourished children interviewed) compared to the size of the sample of the first part of the study (163 malnourished). This is the main limitation of our study, which is why we considered carrying out a simple descriptive work on the questionnaires, which however provides us with a fairly representative picture of the socio-economic and family context in which the malnutrition pathology typically occurs.

## Results

### Sample characteristics

The 163 patients in our sample ranged in age from 6 to 48 months, the median was 12 months, with most (63.2%) in the 6–12 months range, only three patients were over 2 years. There was no significant prevalence of one sex over the other (48.5% male, 51.5% female).

By analyzing the degree of severity of acute malnutrition at admission, indicated by the value of the weight for height z-score (WHZ), it was observed that only 6.8%, showed a moderate acute malnutrition (− 3 SD ≤ WHZ < -2 SD), 47.2% had a severe acute malnutrition (− 4 SD ≤ WHZ < -3 SD), 46% were suffering from very severe acute malnutrition (“Malnutrição aguda muito severa- MAMS”, WHZ < -4 SD). The evaluation of the height for age z-score allowed us to highlight that only 60% of patients had a stunting pattern associated with wasting. In a significant proportion therefore acute malnutrition had arisen in the absence of a previous impairment of linear growth.

The diagnosis of SAM was made for the presence of at least one of the three WHO criteria. In particular only in 7 patients all three criteria were satisfied, in 112 patients the criteria met were MUAC < 11.5 cm and WHZ < -3 SD, in 20 patients only the WHZ criterion, in 11 patients only the MUAC criterion, only one patient had MUAC < 11.5 cm associated with edema, only one was diagnosed for the presence of edema only. So not always all three diagnostic criteria were satisfied.

Another aspect that we wanted to study is the presence of other pathologies or clinical conditions that can sometimes accompany severe acute malnutrition. We do not clearly refer to those acute intercurrent pathologies but to pre-existing clinical conditions, congenital, chronic or long-lasting pathologies.

**Fig. 2:**
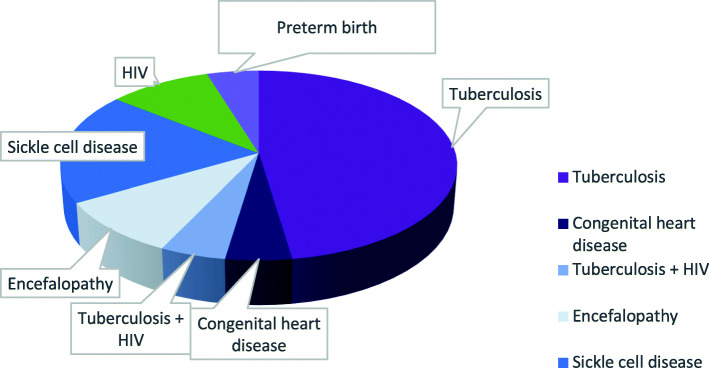
Pathologies or clinical conditions associated with SAM

In our sample, 21 of 163 patients (13%) had the following conditions (Fig. 2): Tuberculosis, HIV, Tuberculosis + HIV, sickle cell disease, encephalopathy, congenital heart disease, preterm birth.

The average length of hospitalization of the patients studied was 7.9 days. If we exclude deceased and escaped patients, the average length of hospitalization for regularly discharged patients is 8.8 days.

The mortality recorded in our sample was 11.7%. In fact, 19 patients died, whose average hospital stay was 3.4 days. In addition, 4 escapes were recorded, a fairly widespread phenomenon that generally concerned very serious patients, with minimal chances of survival outside the hospital. Combining the two groups (deceased and escaped) we have 14.5% of cases in which there has been a therapeutic failure.

### Analysis of potential prognostic factors

We first divided the population into two groups based on age. The first group (103 patients), was aged between 6 and 12 months and with a similar representation of both sexes, the second group (60 patients) aged> 12 months and higher prevalence of females (55% F, 45% M). The variable “age” was not related to a statistically significant difference in the severity of malnutrition at admission or to a different prognosis in terms of length of hospitalization, weight gain and mortality.

We then divided the population into two groups based on the variable “sex”, we saw that males and females showed slight differences in terms of average age (slightly lower in males, 13.5 months vs 14.6) and severity of malnutrition at admission (average WHZ − 4.3 SD in males, − 3.7 SD in females). Even in this case, however, the variable considered (sex) was not significantly associated with a difference in terms of outcomes. Only minimal differences emerged regarding the outcomes considered, but were not statistically significant in the Student’s T test. Mortality was also similar in the two groups (11.4% M, 11.9% F).

Another variable analyzed, as a potential prognostic factor, is the severity of acute malnutrition at the time of admission, represented by the WHZ. We have divided the population into three severity groups: patients with moderate acute malnutrition or MAM (− 3 SD ≤ WHZ < -2 SD); patients with severe acute malnutrition or SAM (− 4 SD ≤ WHZ < -3 SD); patients with very severe acute malnutrition or MAMS (WHZ < - 4 SD). (Table 1).

**Table 1:** Anlalysis of the variable “Severity of acute malnutrition at the time of admission”

Severity of acute malnutrition	Average age	Associated pathologies	Lenght of hospitalization^a^	Average weight gain^a^	Difference target weight/ discharge weight^a^	Difference MUAC at admission/ MUAC at discharge^a^	Mortality
**Moderate (MAM)** **11 patients**	12.8 months	27%	6.2 days	12.3 g/Kg/die	−30 g	0.3 cm	18%
**Severe (SAM)** **77 patients**	14.3 months	8%	8.2 days	14.7 g/Kg/die	180 g	0.4 cm	5.2%
**Very severe (MAMS)** **75 patients**	14.3 months	16%	10.3 days	16.3 g/Kg/die	640 g	0.7 cm	17%

^a^Deaths and escapes were excluded from the average

**Fig. 3:**
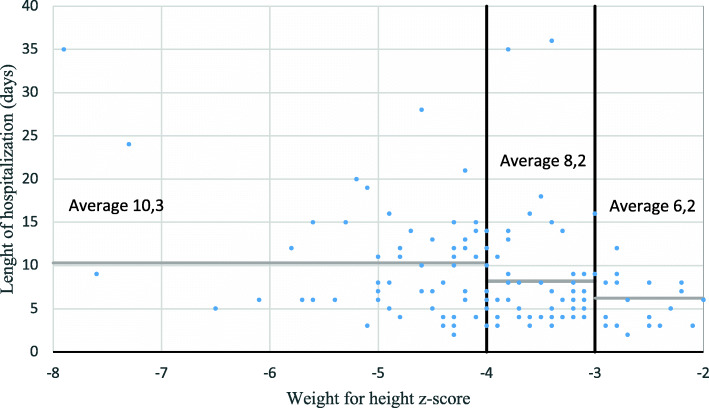
Length of hospitalization in relation to the WHZ value at admission

As shown in Table 1, there are some significant differences between these three groups of patients, especially regarding the presence of pathologies/clinical conditions associated with malnutrition. Furthermore, observing the analyzed outcomes, as the severity of the pathology at entry increases, the average length of stay increases (Fig. 3).

The comparison between the average length of hospitalization of the three groups of patients was made using the Student’s T test. The comparison between the MAMS group and the MAM group allowed us to demonstrate the existence of strong evidence against the hypothesis 0 that the averages are equal (*p* value 0.01). So, the difference between the average length of hospitalization of the MAMS group and the MAM group is statistically significant. However, this difference was not as significant in the comparison between the SAM group and the MAMS group (*p*-value 0.53), nor between the SAM group and the MAM group (p-value 0.18).

The average weight gain of most patients is concentrated in the range between 0 and 20 g/kg/day, but the cases of greater average weight gain (> 20 g / kg / day) mainly concerned those patients starting from a lower WHZ. The application of Student’s T test in this case, however, revealed that the differences are not statistically significant. Probably a greater number of the sample could have confirmed the significance of this difference, especially in the comparison between the MAMS group and the MAM group, which is the one that reported the lowest *p* value (0.18).

**Fig. 4:**
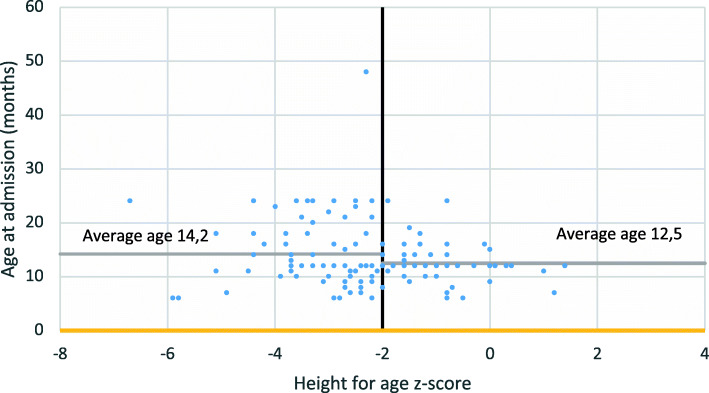
Age of the patients in relation to height for age z-score

We also analyzed whether and how the coexistence of a state of chronic malnutrition (stunting) can influence the prognosis of patients suffering from acute malnutrition. Some differences between patients with and without stunting, emerged regarding the average age, the severity of malnutrition at admission and the prevalence of associated diseases (Table 2, Fig. 4). The analysis of the outcomes related to growth showed that patients with stunting grow on average more during hospitalization, but the Student’s T test did not give us a value of statistical significance (*p*-value 0.26). The mortality was instead significantly higher in the stunting group than in patients without stunting.

**Table 2:** Analysis of the variable “presence of stunting”

Lenght for age	Average age	Average WHZ at admission	Associated pathologies	Lenght of hospitalization^a^	Average weight gain^a^	Difference target weight/ discharge weight	Difference MUAC at admission/ MUAC at discharge^a^	Mortality
**< −2 SD** **(Presence of stunting)** **98 patients**	14.2 months	−4.2 SD	17%	8.9 days	15.9 g/Kg/die	260 g	0.6 cm	13.3%
**≥ − 2 SD** **(Absence of stunting)** **65 patients**	12.5 months	−3.9 SD	6%	8.4 days	13.5 g/Kg/die	460 g	0.4 cm	9.2%

^a^Deaths and escapes were excluded from the average

By subdividing the population based on the presence or absence of associated pathologies, substantial differences emerged both in terms of individual characteristics and in prognostic terms (Table 3). Regarding the average age, the application of the Student’s T test allowed us to demonstrate that this difference is statistically significant (*p*-value 0.007).

**Table 3:** Analysis of the variable “presence of associated pathologies”

	Average age*	Average WHZ at admission	Presence of stunting	Lenght of hospitalization^a^	Average weight gain^a^	Difference target weight/ discharge weight^a^	Difference MUAC at admission/ MUAC at discharge^a^	Mortality
**Presence of associated pathologies** **21 patients**	16.7 months	- 4.7 SD	80.9%	14.89 days	7.94 g/Kg/die	490 g	0.6 cm	0
**Absence of associated patgologies** **142 patients**	12.9 months	−3.9 SD	55.6%	7.74 days	16.29 g/Kg/die	320 g	0.4 cm	13.4%

^a^Deaths and escapes were excluded from the average

Patients with associated diseases had a longer average length of hospitalization and a lower average weight gain. Also in these cases it has been shown that the difference between the averages is statistically significant, being the p-value 0.007 in the case of the average weight gain, even lower in the case of the duration of hospitalization (< 0.0001).

### Analysis of potential risk factors

We first evaluated the characteristics of the sample of 52 malnourished whose mothers were interviewed, to verify that it was representative of the entire malnourished population. The average age of this group was 12 months, 46.2% were male, 53.8% female, the mean WHZ in the interviewed sample was − 3.9 and the mean MUAC was 10.6. For all these aspects, the sample studied could be considered quite representative of the UEN patient population. The only data that differs is that of mortality, which is lower in the sample of the 52 patients interviewed (5.8%).

The average age of the sample of 23 non-malnourished children, whose mothers were interviewed, was 14.6 months, the median of 12 months. 52% were male children, 48% female. Only one of these children died.

In perinatal anamnesis, the first aspect that we have analyzed is access to health services. We investigated the place of birth (home or hospital), the execution of antenatal visits and vaccinations. In both groups most patients were born in hospital or health center, had vaccinations and most mothers had visits during pregnancy (Table 4).

**Table 4:** Access to health services

Groups	Place of birth				Antenatal visits				Vaccinations			
	*Home*		*Hospital/ Health center*		*Done*		*Not done*		*Done*		*Not done*	
	N.	%	N.	%	N.	%	N.	%	N.	%	N.	%
**Patients admitted to UEN**	22	42	30	58	45	86.5	7	13.5	48	92	4	8
**Patients admitted to pediatrics**	11	48	12	52	23	100	0	0	22	96	1	4

Another risk factor analyzed is birth weight, which however cannot always be known precisely. In fact, there is no habit of memorizing the birth weight of children and almost always there is no health document to certify it. As the exact birth weight was often not known, mothers were asked if their baby at birth was very small or normal/large. Although we are aware of the considerable approximation of this data, differences between the two groups emerged in this regard. Among the mothers of malnourished patients, 54% reported that their child was born large or normal, 42% that it was born very small, 4% did not know. Among the mothers of the non-malnourished, 91% reported that their child was born large or normal, 9% that they were born small. Being born small could indicate prematurity or low weight for gestational age and could be a sign of maternal malnutrition [11].

The next part of the questionnaire was based on the patient’s eating habits with particular regard to breastfeeding and introduction of complementary foods. Table 5 shows that critical issues emerged in this regard in both groups, but with some differences (Table 5).

**Table 5:** Patient’s eating habits

Group	Age of introduction of water		Age of suspension of breastfeeding				Interval between suspension of breastfeeding and hospitalization			Age of introduction of complementary foods			
	< 6 months	> 6 months	Not suspended yet	< 6 months	6–12 months	> 12 months	< 3 months	3–6 months	> 6 months	Not introduced yet	< 3 months	3–6 months	> 6 months
**Patients admitted to UEN** **(malnourished)**	65%	35%	35%	4%	27%	33%	42%	11.5%	10%	2%	25%	23%	50%
**Patients admitted to pediatrics (not** **malnourished)**	70%	30%	70%	0	13%	17%	4%	4%	22%	–	13%	39%	48%

The WHO in 2008 published the document “Indicators for assessing infant and young child feeding practices” in which it describes indicators for the evaluation of infant and child nutrition, among these it introduces the concept of minimum acceptable diet - MAD), an index composed of the “minimum dietary diversity” and the “minimum meal frequency” for children aged between 6 and 23 months [12].

The criterion of minimum dietary diversity (intake of foods belonging to at least 4 different groups) in the group of malnourished patients was met in only 33% of cases. On average the patients in this group ate foods belonging to 2.8 different groups. If we consider the availability of food for the family unit, however, it emerges that 79% of the mothers interviewed report having food available at home belonging to at least four different groups. In the group of non-malnourished patients, 65% meet the criterion of minimum dietary diversity, with an average of 3.7 different food groups. Even in this case, however, there is a difference with the percentage of families who have food from at least 4 different groups (83%). Mothers were also interviewed about the type of food their children eat (Table 6).

**Table 6:** Percentages of patients who eat the foods of the different categories, in the UEN group and in the Pediatrics group

Food category	Patients admitted to UEN	Patients admitted to Pediatrics
Cereals, roots and tubers	96%	91%
Legumes and nuts	31%	52%
Dairy products	42%	52%
Flesh foods	42%	52%
Eggs	27%	57%
Fruits and vegetables	35%	61%

The other parameter that constitutes the “minimum acceptable diet” is the “minimum frequency of meals”, which is different in breastfed than in non-breastfed children and in relation to age. For breastfed babies the minimum number of solid/semi-solid meals should be 2 per day between 6 and 8 months, 3 per day between 9 and 23 months. For non-breastfed infants, the minimum frequency of meals (solid/semi-solid and in this group also milk-based meals) is 4 between 6 and 23 months. This criterion was met among the malnourished in only 4 patients out of 52 (7.7%) and all these 4 children were breastfed. In the group of non-malnourished patients, 4 out of 23 met this criterion (17.4%), also in this case they were all children still breastfed.

Patients who meet both WHO criteria (minimum frequency of meals and minimum dietary diversity) are 2 out of 52 among the malnourished (3.8%) and 3 out of 23 among the non-malnourished (13%).

To conclude our analysis on risk factors for malnutrition, we analyzed family risk factors, with particular regard to maternal ones.

First was asked if the child’s caregiver was the mother or another figure (usually another female family member). In the malnourished group, 83% of children lived with their mother, 17% with another family member, often because they were orphans. Among the non-malnourished, 95.5% lived with their mother, 4.5% did not.

Among the potential maternal risk factors, we considered age, education, BMI and the presence of HIV and Tuberculosis.

In the malnourished group, 13.5% of mothers were under the age of 20, 50% were between 20 and 30, 17.3% between 30 and 40 and 19.2% had more than 40 years. The group of mothers of non-malnourished patients did not show great differences in terms of age (13% < 20 years, 65% between 20 and 30, 9% between 30 and 40 and 9% > 40, 4% age not known).

Another factor considered is the maternal education, also in this case the two samples were comparable in both cases being constituted by approximately 50% of mothers who had attended the school and 50% of mothers who had never attended the school.

Regarding the BMI, no relevant differences emerged between the two groups. In both groups, most of the mothers were of normal weight (BMI between 18.5 and 25).

With regard to the presence of associated pathologies, in the malnourished group one of the mothers was infected with HIV and had died, another was suffering from tuberculosis. None of the mothers in the non-malnourished group presented these pathologies.

To analyze the characteristics of the family unit, we asked the mothers how many children they had and if all were still alive or, if not, how many of them had died. On this aspect, important differences emerged between the two groups.

On average, the mothers of the malnourished had 4.2 live children compared to 2.7 for the mothers of the non-malnourished. Malnourished children therefore seem to belong on average to larger families. However, the even more striking figure concerns the deceased children. Out of 52 mothers of malnourished patients interviewed 23 (44%) had had at least one deceased child (among these the average was two deceased children each). In the other group, however, 3 mothers out of 23 (13%) had lost at least one child.

Finally, we tried to analyze some characteristics that could be indicative of the socio-economic level of the family unit. We considered the type of house and the type of kitchen in which the meals were prepared. We asked the mothers if they lived in a hut (71% of the malnourished vs 43% of the non-malnourished), in a house made of sheet metal (17% of the malnourished vs 43% of the non-malnourished) or in a house made of cement (12% of malnourished vs 9% of non-malnourished). With regard to the type of cuisine used, 90.4% of the mothers of the malnourished and 91.3% of those of the non-malnourished cooked with wood, 9.6% of the former and 8.7% of the latter with a gas kitchen.

## Discussion

From the analysis of the characteristics of our sample, it emerged that hospitalizations for acute malnutrition at the Chiulo Hospital, in accordance with the literature data, mainly concern children aged between 6 and 24 months. This is the age group most susceptible to this disease. This period corresponds with the introduction of complementary foods and unfortunately, very often with an early suspension of breastfeeding [13]. Before 6 months, breastfeeding plays an important protective role, none of our patients were less than 6 months old.

Regarding the diagnostic criteria for SAM, our data confirmed the importance of assessing all the parameters to prevent some cases might escape diagnosis. In fact, not always all three diagnostic criteria were met [14, 15]. In our sample, 21 patients would have escaped the diagnosis with the sole use of MUAC (a widespread practice especially in peripheral centers).

The mortality of our sample was quite high, but similar to that reported in the literature [16]. Most of the deceased were patients who already arrived in extremely serious conditions. The death often occurred a few days after hospitalization, as demonstrated by an average length of hospitalization significantly shorter than the rest of the sample. This data is consistent with those reported in similar studies carried out in other countries [17].

The analysis of prognostic factors has shown that the variables “age” and “sex” do not seem to be associated with significant changes in the clinical outcome. Differences in terms of outcome were instead observed among patients with different degrees of severity at the time of hospitalization as shown in Table 1. In fact, the most serious patients, in terms of WHZ at admission, remain hospitalized longer but seem to grow more both in terms of weight and MUAC (even if the small number of the sample does not allow us to confirm the latter data). Mortality was much higher in the group of patients with lower WHZ. This is probably the data that more clearly confirms that the lower is the WHZ, the worse is the prognosis. Mortality in the MAM group is also high, but this assumes little significance in relation to the low number of the sample and the high percentage, in this group, of patients with associated diseases.

Furthermore, among the most serious patients the percentage of those with associated diseases was higher. This leads us to reflect on the importance of always suspecting an associated disease, especially in those with a WHZ < - 4 DS.

Another variable considered as a possible prognostic factor is the presence of stunting. It should be emphasized that the height for age z-score, in rural contexts such as Chiulo, has an important limit. Most children are not registered at birth and do not present any documents [18]. Furthermore, the date of birth is not given the importance that we are accustomed to attribute to it. So the mothers often did not remember the date of birth of their children. It follows that the reported age did not always correspond to the real one. Any evaluation on the height for age parameter must take this aspect into account. However, we considered it appropriate to make an assessment of chronic malnutrition, while not ignoring these limits.

Regarding the variable “presence of stunting”, we observed that the average age is higher in the stunting group as well as the length of hospitalization; in both cases, the difference is not statistically significant. However, a fact to be highlighted is that the very few cases of long-term hospitalizations (over 30 days) are all concentrated in the stunting group. These are patients suffering from diseases associated with SAM, mainly tuberculosis, which justify the longer length of hospitalization and which typically, also compromise linear growth. The presence of associated pathologies and mortality were much higher in the stunting group.

The variable that gave the most significant results is the presence of associated pathologies. Patients with associated pathologies were on average older. Three of these 21 patients were even older than 24 months, and were therefore outside the typical age range for developing SAM. This confirms that in an older patient with SAM it is always important to look for an associated pathology. The “primitive” SAM is typically a pathology of the younger child.

In addition, patients with associated pathologies had a WHZ lower at the admission, they more often presented stunting, and this is easily to understood by knowing the impact that chronic diseases have on linear growth [19]. They remained hospitalized longer and had lower average weight gain. All these differences were statistically significant. A data in contrast with these is that of mortality, no deaths were recorded in the group of patients with associated pathologies. However, it should be considered that the deaths all occurred in the first 2-3 days. So, we cannot exclude that among the deceased there were patients with associated diseases that we did not have time to diagnose.

The second part of our study focused on identifying potential risk factors for acute malnutrition. Due to the time required for the administration of the questionnaire and the need for local staff to act as interpreters, the final number of questionnaires was quite limited. The very small number of mothers of non-malnourished patients interviewed is sadly linked to the very high prevalence of malnutrition in this geographical area. Often, even patients hospitalized in Pediatrics for other pathologies, presented some degree of malnutrition that did not allow us to include them in the control group. However, despite the limitations linked to the low number of samples, we believe that our data shows a fairly representative picture of the context in which malnutrition occurs.

We first evaluated if the sample of malnourished was representative of the entire population of patients admitted to UEN. The two samples were comparable for most of the characteristics except for mortality. This is influenced by the fact that we have rarely had time to administer the questionnaire to the mothers of the deceased children. The short time of hospitalization, together with the extremely serious conditions of these patients, did not allow creating the conditions for the administration of the questionnaire.

The first aspect we analyzed is access to health services, potential opportunities for health education. In theory, those who have had more opportunities to access services should be more sensitive to certain issues (such as malnutrition) than those who have never or almost never had recourse to a health facility (quite frequent occurrence in such contexts). Any access to services should be an opportunity to carry out health education with particular reference to nutritional education. Our data did not reveal any significant differences between the two groups, in both there had been occasions of access to services. It therefore emerges that access to health services is likely to be opportunities that are not exploited enough. It would be advisable to carry out awareness campaigns and training of health personnel on this aspect. This is an intervention almost at no cost that could have important implications. At the Chiulo Hospital, patients in the waiting room are entertained with the so-called “gyms”, that is short and simple lessons by the staff on certain pathologies and their prevention strategies. This habit should be extended to the other wards and health centers.

The analysis of eating habits revealed interesting data, especially on breastfeeding and weaning habits. Breastfeeding is known to be protective against numerous pathologies, for its ideal nutritional characteristics but also for the countless other properties, first of all the immunomodulating ones [20, 21]. In developing countries, its role becomes even more important as it can really make the difference between the survival and death of children [5, 9]. It is the only food that is safe in microbiological terms in the first months of life (scarce availability of safe water sources). In many contexts, it is the only one that can guarantee the nutrients necessary for a growing organism, in consideration of the very limited availability of other foods. It is for these reasons that, despite the small number of our samples, substantial differences emerge between the malnourished group and the non-malnourished group regarding breastfeeding.

Almost none of both groups had suspended breastfeeding before 6 months, but a significant proportion of the malnourished had suspended it between 6 and 12 months. It is also significant that at the time of admission only 35% of the malnourished were still breastfed compared to 70% of the non-malnourished, despite an average age comparable in the two groups. This may be indicative of the protective role of breast milk against SAM. One element that we wanted to investigate is the interval between the suspension of breastfeeding and hospitalization for SAM to highlight a potential role of the suspension of breastfeeding as a trigger for acute malnutrition. Among patients admitted to UEN, 42% had stopped breastfeeding less than 3 months before admission. In these children it is likely that this dietary change was the trigger for SAM. Of the patients admitted to pediatrics (not malnourished), only 4% had stopped breastfeeding less than 3 months earlier.

There was a fairly low prevalence of exclusive breastfeeding in the first 6 months in both groups. In this regard, the analysis of habits related to the weaning revealed critical issues in both groups. Among the malnourished, even 25% had started taking complementary foods before 3 months of life. The extremely early introduction of complementary foods is confirmed to be an important risk factor for the development of malnutrition, as already highlighted by other studies [22]. When other foods are introduced, the intake of breast milk and therefore its production is inevitably reduced. In addition, the risks of administering semi-solid foods to an individual not yet mature enough to take them, together with the poor quality of the foods administered, contribute to the extreme inadequacy of nutrition that these children practice. Another aspect studied is the age of introduction of the water. In the context in which our study was carried out, the administration of water under the age of 6 months was an extremely widespread practice (as shown in Table 5). In such contexts, the recommendation not to give water to small infants should be even stronger. In fact, water is often unsafe and can seriously endanger the survival of these children.

Therefore, despite the high prevalence of breastfeeding (almost 100% of children have practiced it), are absolutely unsatisfactory the percentages of those who only take breast milk in the first 6 months of life, introduce complementary foods at the correct times and continue breastfeeding up to 24 months [3].

With regard to the type of food consumed, there was little dietary variability in both groups. An interesting fact is the discrepancy between the availability of food at home and its intake by children. Often at the basis of inadequate nutrition, there is not only limited food availability but also cultural factors and lack of knowledge of children’s needs [23]. It is clear once again how important it is to carry out nutrition education campaigns to educate families on how to make the most of available resources [24]. The foods in the category of cereals, roots and tubers are the most widely consumed. Many families grow various types of cereals to produce flours that they use in the preparation of “funge” (a staple food typical of Angola). There are many children, especially in rural areas, whose diet consists almost exclusively of foods of this group with consequent very serious nutritional deficiencies. They are low-cost, locally produced foods that easily give a sense of satiety, thus allowing, with a minimum expense, to feed very large families. Foods rich in proteins (legumes, dairy products, meats, eggs) are consumed only by small percentages of children, especially among the malnourished. False beliefs also play their role, such as the belief that that children cannot take eggs or that legumes cause diarrhea. Furthermore, there is no habit of transforming foods such as meat and fish to prepare baby food that also little children can eat. The percentages of those who meet the minimum meal frequency criterion are also very low. In particular, none of the children not breastfed in both groups meets this criterion. It once again emerges that breastfeeding is a fundamental resource for guaranteeing children of this age group a diet that is minimally acceptable, even in the presence of extremely limited economic resources.

The analysis of family risk factors revealed a greater risk of malnutrition in those who do not live with their mother. This may partly be because they are often orphans, who have never been breastfed or have been for a short time.

Among the family risk factors, age, educational level and BMI of the mother, according to our data, do not seem to be significantly associated with childhood malnutrition, although the small number of samples does not allow us to exclude a correlation, which has instead been demonstrated in other studies [7, 25].

The differences in the characteristics of the family unit were relevant. On average, the families of the malnourished were more numerous. The percentage of families in which at least one child had died was higher in this group, although even among the non-malnourished this percentage was high. In both cases, these are dramatic numbers, especially when compared with our realities in which, the loss of a child is an exceptional event. However, once the emotional impact of such data has been overcome, it is evident that the presence of deceased children always represents an important alarm bell that should never be underestimated and that must make us consider that patient at high risk.

Regarding the two indices of socio-economic level considered (type of house and type of kitchen) the first was different in the two groups (with the vast majority of malnourished people living in a hut), the second was instead comparable in the two groups. We believe that more numerous samples would probably have confirmed a correlation between the risk of malnutrition and the socio-economic level, as already demonstrated in other studies.

## Conclusions

This work, despite the limitations relating to the short period in which the data collection could be carried out and consequently to the small size of the population studied, wanted to provide a picture of the main issues still open in the field of acute malnutrition.

In some rural contexts, such as the one examined, the malnutrition rate is so high that the vast majority of children, especially under 2 years of age, have some degree of malnutrition. So, in the eyes of their parents, those children seem “normal” when compared to others of the same age [19]. The perception of the malnutrition problem often by caregivers is absolutely non-existent, until one acute event occurs that precipitate the delicate balance that had been created, leading to a full-blown picture of severe acute malnutrition.

Based on the collected data, some interesting elements emerged regarding the risk factors for the development of the disease, the diagnosis of acute malnutrition, but also regarding potential prognostic factors. Our data have confirmed the importance, in the face of a child with suspected malnutrition, to carry out a global clinical evaluation using all the anthropometric measures available to prevent many cases from escaping diagnosis and treatment. In this regard, staff training programs would be essential for identifying and managing cases of malnutrition, especially in more peripheral contexts [26].

Among the prognostic factors, the most relevant were the WHZ at the time of admission, the presence of stunting and the presence of chronic diseases/clinical conditions associated with SAM.

A lower weight for height z-score (WHZ) correlate with longer length of hospitalization, higher mortality, and, in those who survived, a trend towards greater average weight gain.

Patients with Stunting were older on average, had associated diseases more often, and had slightly higher mortality.

Patients with other associated pathologies/conditions had a higher average age than the others, a longer average length of hospitalization and a lower average weight gain. SAM associated with other pathologies has very different characteristics compared to “primitive” SAM, often the underlying pathology is the main etiological factor of acute malnutrition. While such patients tend to have a more severe disease course, on the other hand if the disease in question is promptly identified and treated (where this is possible as in the case of tuberculosis), the chances of recovery from SAM are much higher.

With regard to risk factors, the importance of breastfeeding as a protective factor against malnutrition has emerged in a striking way.

Even the practice of weaning is often burdened by significant problems and often this is not due solely and simply to the food shortages. Nutritional education campaigns are necessary, they should be based on a deep knowledge of the socio-economic and cultural context in which the patients live, of the available resources and they should be carried out on the territory. Even when these efforts are carried out flawlessly, there are, however, a considerable number of cases in which the basic problem remains the dramatic unavailability of nutrients. So, the best impact is likely to be achieved for those interventions in which the provision of complementary foods is combined with nutrition education [13].

Malnutrition is a multifactorial pathology in which genetic, epigenetic, cultural, environmental, socio-economic factors interact with each other. Unfortunately, it is a pathology that is little known in developed countries but which, due to the dramatic impact it has on children’s health worldwide, deserves to be known at all latitudes. In today’s world, in fact, having a projected look on global health is now necessary for those involved in health care. Opening up to health problems that have historically been considered of exclusive interest to “distant” countries, is a precious opportunity not only for those who want to spend themselves in the field of international cooperation, but also for those involved in child health in our country, always more multi-ethnic and globalized.

## Data Availability

The datasets used and/or analyzed during the current study are available from the corresponding author on reasonable request.

## Abbreviations

BMI: Body Mass Index
CUAMM: Collegio Universitario Aspiranti Medici Missionari
HIV: Human Immunodeficiency Virus
IIMS: Inquérito de Indicadores Múltiplos e de Saúde
MAM: Moderate Acute Malnutrition
MAMS: “Malnutrição aguda muito severa” (Very severe acute malnutrition)
MUAC: Mid-upper arm circumference
NGO: Non Governmental Organization
RUTF: Ready to Use Therapeutic Foods
SAM: Severe Acute Malnutrition
UEN: “Unidade Especial de Nutrição” (Special Nutrition Unit)
WHO: World Health Organization
WHZ: Weight for height z-score
